# Grape Cluster Detection Using UAV Photogrammetric Point Clouds as a Low-Cost Tool for Yield Forecasting in Vineyards

**DOI:** 10.3390/s21093083

**Published:** 2021-04-28

**Authors:** Jorge Torres-Sánchez, Francisco Javier Mesas-Carrascosa, Luis-Gonzaga Santesteban, Francisco Manuel Jiménez-Brenes, Oihane Oneka, Ana Villa-Llop, Maite Loidi, Francisca López-Granados

**Affiliations:** 1 Grupo Imaping, Instituto de Agricultura Sostenible-CSIC, 14004 Córdoba, Spain; fmjimenez@ias.csic.es (F.M.J.-B.); flgranados@ias.csic.es (F.L.-G.); 2 Departamento de Ingeniería Gráfica y Geomática, Campus de Rabanales, Universidad de Córdoba, 14071 Córdoba, Spain; ig2mecaf@uco.es; 3 Departamento de Agronomía, Biotecnología y Alimentación, Universidad Pública de Navarra, 31006 Pamplona, Spain; gonzaga.santesteban@unavarra.es (L.-G.S.); oihane.oneka@unavarra.es (O.O.); ana.villa@unavarra.es (A.V.-L.); maite.loidi@unavarra.es (M.L.)

**Keywords:** fruit detection, remote sensing, precision viticulture, color thresholding, unsupervised and automated analysis

## Abstract

Yield prediction is crucial for the management of harvest and scheduling wine production operations. Traditional yield prediction methods rely on manual sampling and are time-consuming, making it difficult to handle the intrinsic spatial variability of vineyards. There have been significant advances in automatic yield estimation in vineyards from on-ground imagery, but terrestrial platforms have some limitations since they can cause soil compaction and have problems on sloping and ploughed land. The analysis of photogrammetric point clouds generated with unmanned aerial vehicles (UAV) imagery has shown its potential in the characterization of woody crops, and the point color analysis has been used for the detection of flowers in almond trees. For these reasons, the main objective of this work was to develop an unsupervised and automated workflow for detection of grape clusters in red grapevine varieties using UAV photogrammetric point clouds and color indices. As leaf occlusion is recognized as a major challenge in fruit detection, the influence of partial leaf removal in the accuracy of the workflow was assessed. UAV flights were performed over two commercial vineyards with different grape varieties in 2019 and 2020, and the photogrammetric point clouds generated from these flights were analyzed using an automatic and unsupervised algorithm developed using free software. The proposed methodology achieved R^2^ values higher than 0.75 between the harvest weight and the projected area of the points classified as grapes in vines when partial two-sided removal treatment, and an R^2^ of 0.82 was achieved in one of the datasets for vines with untouched full canopy. The accuracy achieved in grape detection opens the door to yield prediction in red grape vineyards. This would allow the creation of yield estimation maps that will ease the implementation of precision viticulture practices. To the authors’ knowledge, this is the first time that UAV photogrammetric point clouds have been used for grape clusters detection.

## 1. Introduction

In the last few years, grape growers have made substantial investments for optimizing grape production and wine quality—e.g., vertical-shoot positioned trellised vines, mechanical harvest, drip-irrigation, or even fertigation—and often cropped under no-tillage techniques or with natural or sown cover crops in inter-rows for overcoming soil erosion problems. Accurate yield estimation is essential for grape growers, viticulturists, and winemakers interested on digitizing applications and precision viticulture, since they can plan harvest operations in advance, or apply a variety of site-specific management practices such as leaf removal or cluster thinning to improve fruit ripening process [1]. Additionally, from the winery logistic point of view, a sufficiently accurate yield forecast can be crucial to anticipate grape purchase to external suppliers in order to meet the winery needs if forecast production is below them, or to intensify sale operations of the wine stock if yield predictions overcome the demand. In this regard, the key point is that a profitable and accurate yield forecasting requires timely, detailed, individual, and georeferenced information of every vine acquired at the lowest possible cost. In addition, the procedure must be easily repeated on demand annually. Since yield can significantly vary locally and globally from year to year due to soil/climatic/management conditions, and eventually due to sanitary problems caused by pests, weeds, and diseases, among others [2,3].

All these economic and agronomic factors make yield estimation challenging and, due to its relevance, is has been addressed from several different approaches. Traditional methods trust on-ground measurements, performed usually with a small sample of selected vines, where bunches are weighed and counted providing the average number of clusters per vine and the average weight per cluster. This information was extrapolated to the whole vineyard knowing the number of vines per hectare. This methodology is destructive and time-consuming, and often inaccurate and inefficient due to poor sampling representativeness [4].

One of the alternative approaches could be imaging-based developments focusing on the use of proximal or remote imagery. The first of these approaches generally uses a manual acquisition of images, a frame equipped with a sensor, or on-the-go human-driven or robotic vehicles. The second strategy needs imagery from satellite, piloted, or unmanned aerial platforms. Considering proximal sensing, visible spectrum cameras have been employed for grape berry recognition and grape bunch detection by shape and texture information [5]. That work also evaluated the existing methods and comparative analysis of different feature vectors and support vector classifiers. The application of on-ground digital image analyses for grape detection covering the key challenges for ground level image capture and processing has been reviewed [3]. Kinect RGB-D sensor and cameras incorporated on a vehicle driving through the vineyard have been used for in-situ vineyard yield estimation [6,7]. These works discussed the relevance of both, imaging and algorithmic robustness for maintaining consistent performance of image analysis methods, suggesting that image acquisition at night was the most reliable way to control the imaging. That night condition has been employed [8] taking images ‘on-the-go’ of the vine segments in a set of varieties using a RGB sensor-equipped all-terrain vehicle with a white-light LED panel. All these works have some limitations for reliable yield estimation related to a possible slope, a plowed or wet soil, or presence of cover crops in the vineyard that can cause measurement difficulties and the possibility to generate efficient and robust results at a parcel scale.

In case of remotely sensed imagery, the UAV (unmanned aerial vehicle) is being increasingly used for accurately monitoring of crops [9,10,11] due to its very high spatial and temporal resolution, low cost, easy access to difficult zones and absence of soil compaction. All these characteristics make UAV technology an adequate tool for mapping perennial crops such as almond [12,13], olive [14,15], or vineyards at the field scale for different proposes related with—e.g., 3D canopy characterization, water stress, or site-specific weed control [16,17,18,19,20,21,22]. As happens with on-ground images, one of the most challenging objectives for UAV image analysis is to develop cost-effective, robust and straightforward repeatable procedures for vineyard yield estimation at field scale. An automated estimation of yield has been obtained [23] by determining cluster number and size from individual non-mosaicked UAV-high resolution RGB images according to vigor variability and considering partially leaf removal and level of ripeness at the time of image acquisition. An assessment of the spatial variability of vegetative, productive, and berry composition using a set of derived spectral indices from multispectral-UAV imagery showed no significant correlations between yield and the studied spectral indices [24]. Other authors have applied artificial neural networks to multispectral UAV imagery to model the relationship between vegetation indices, vegetated fraction cover, and yield referring RMSE = 0.5 kg∙vine^−1^ [25].

The use of photogrammetric techniques applied to RGB-UAV-images for generating point clouds has been reported as a very efficient method to geometrically and accurately characterize a vineyard for supervising leaf removal and other canopy management operations [20]. Point clouds representing vine rows include a huge amount of information that must be processed together to advanced and automated image analysis procedures. Since together to the geometry, each point also collects information from the RGB color model, point clouds can be accurately classified into non-vegetation (trunks and branches of vines) and vegetation classes using a set of spectral indices for discrimination of vegetation points for further assessment of the height of vines [26].

However, to the best of our knowledge, photogrammetric point clouds generated from UAV imagery have not been applied to grape cluster detection for vineyard yield forecasting. Taking into account that one of the strategies for classifying clusters in proximal sensing has been to extract the color features using manual thresholds and tolerances for the segmentation [27], conversely an automatic imaging-based development for UAV imagery taking advantage of the color from the points could be also studied. This is the differential and innovative aspect of the present work, aimed to show a new method for classifying 3D-UAV photogrammetric point clouds using RGB information through a color filtering process for cluster detection. This procedure was evaluated in two vineyards over two years using low-cost tools such as an UAV equipped with a RGB inexpensive sensor and free software for image analysis. Our specific objectives included (1) determining the relationship between the points classified as grapes and the harvest weight, and (2) assessing the influence of leaf occlusion in this relationship.

## 2. Materials and Methods

### 2.1. Study Sites and Experiment Description

**Figure 1:**
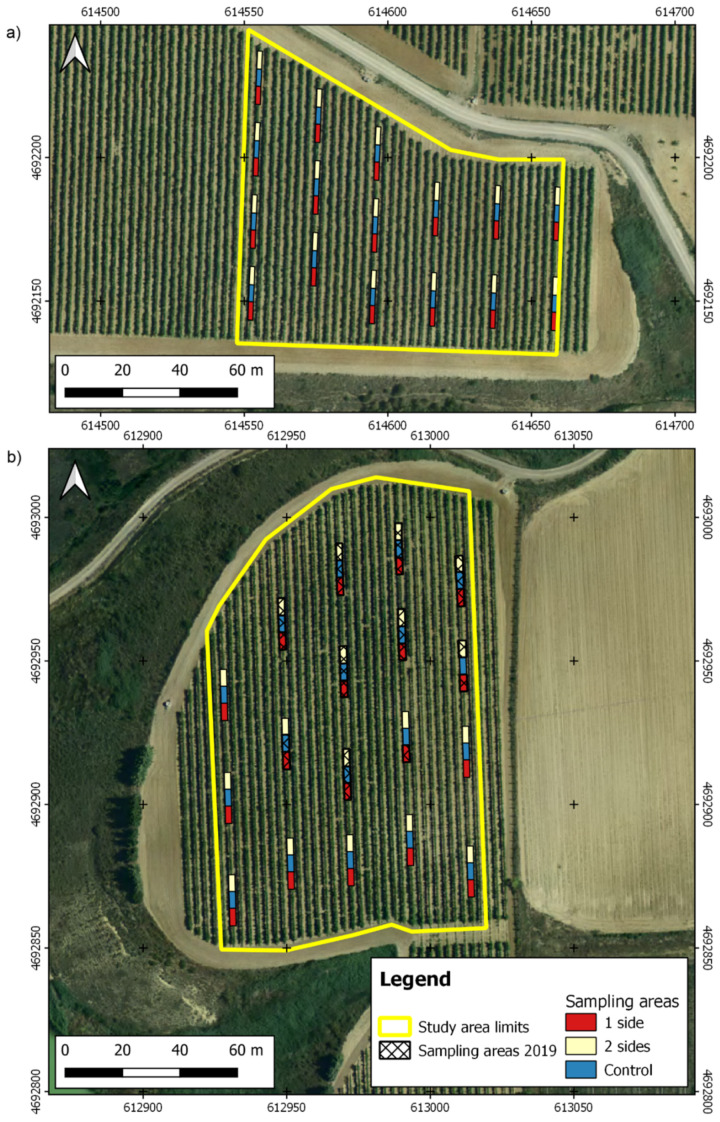
Maps of the study sites and the sampling points: (**a**) field with Garnacha variety; (**b**) field with Graciano variety. Coordinate system: WGS84 UTM30N.

The experiment was carried out in years 2019 and 2020 in a wine farm located in Traibuenas (Navarra, Northern Spain, 42.379° N, 1.621° W, altitude: 335 m), in a region characterized by a semiarid climate (Bs type in Koppen’s classification; *p* < 400 mm; ETPPenman > 1150 mm). Two vineyards were selected for (Figure 1), one belonging to Graciano variety, with a 1.37 ha area, whereas the other had an area of 0.91 ha and belonged to Garnacha tinta (syn. Grenache Noir) variety. The vineyard planted with Graciano was 21-year-old at the beginning of the research, whereas the Garnacha tinta vineyard was one year younger. Both fields had a planting distance of 3 × 1 m with N-S oriented vine rows, and their vines were trellised as a vertical-shoot positioned Cordon de Royat. Both fields locate in ancient terraces of Aragon and Cidacos rivers, and can be classified in the Typic Calcixerept group [28,29]. The variability within each field is moderate, and it is associated to changes in gravel content in Graciano vineyard, and to a moderate slope in Garnacha noir vineyard. In 2019, mean temperature and precipitation were 13.8 °C and 420 mm (18.8 °C and 230 mm in the Apr-Sep period), while in 2020 they were 13.9 °C and 441 mm (19.1 °C and 218 mm between April and September). Drip irrigation was available in both fields, and water was applied according to winery technical staff criteria considering a deficit irrigation strategy. As an average, irrigation accounting for 12 mm wk^−1^ was provided between mid-June (fruit set) and early September.

**Figure 2:**
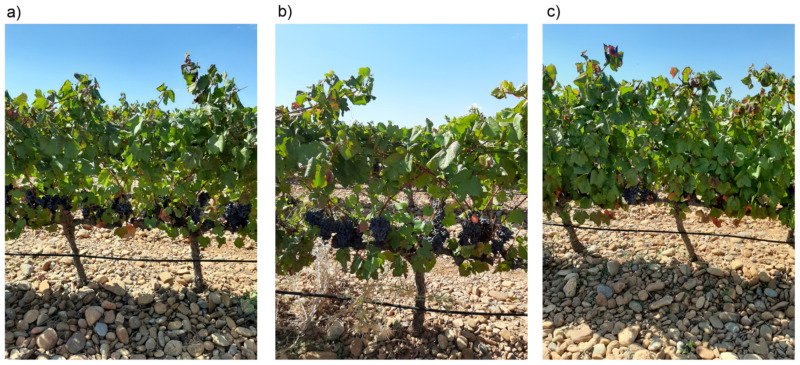
Field photos representing the different leaf removal treatments: (**a**) one side; (**b**) two sides; (**c**) control.

In order to assess the influence of the occlusion by leaves on cluster detection, a set of harvest sampling zones were selected where leaf removal was done in two different modes (Figure 2): leaf removal in the east side of the row, and leaf removal in both sides of the row. The leaves from the basal 40 cm of all the shoots were removed in both treatments, without considering if they belonged to the main shoot or to laterals. In each of the established sampling zones, two sides leaf removal was applied to six vines, followed by six vines that remained untouched as control, and by other six vines where one side leaf removal was applied.

Each one of the sets of 6 vines were manually harvested (harvest dates were 25 September 2019 and 21 September 2020 for Graciano and 14 September 2020 for Garnacha), and their harvest weight was used as validation data for the cluster detection algorithm. A total of 18 sampling sites were established in the Graciano field in 2019 and 2020, while the data from the Garnacha field were collected in 16 sampling sites in 2020. From here on, data from Graciano and Garnacha fields will be abbreviated as Gr-19 and Gr-20, and as Ga-20, respectively.

### 2.2. UAV Flights and Point Cloud Generation

Aerial imagery from the Graciano and Garnacha fields was collected on 8 September 2020, and a previous flight campaign was also executed in the Graciano field on 17 September 2019. Both years, grapes were ripe, and the flights were carried out in sunny days with low wind conditions and around noon.

**Figure 3:**
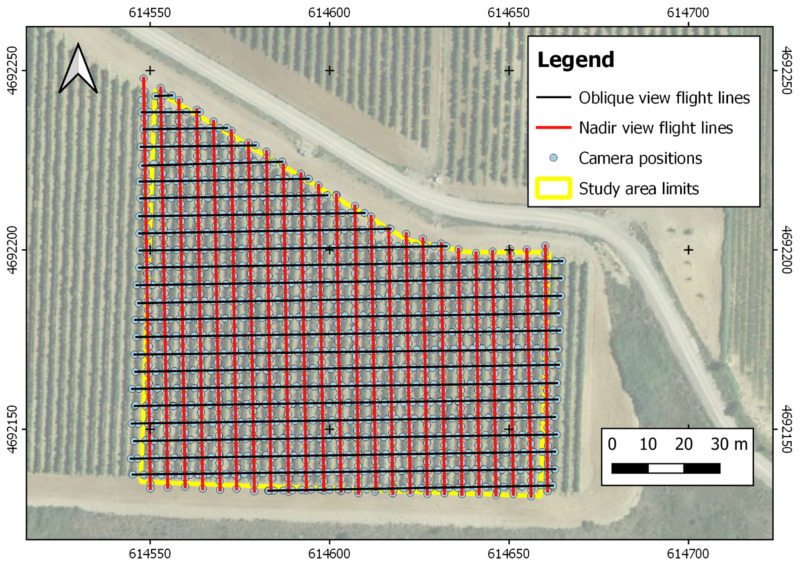
Map of the Garnacha field showing the coordinates of the image acquisition points and the UAV flight lines with the different sensor orientations. Coordinate system: WGS84 UTM30N.

The aerial imagery from Graciano field in 2019 was collected using a Sony ILCE-6000 (Sony Corporation, Tokyo, Japan) camera equipped with 24 MP sensor and a 20 mm fixed focal length lens. The camera was installed on board a quadcopter model MD4-1000 (microdrones GmbH, Siegen, Germany). The aerial images were acquired using two types of flights: one with flight lines parallel to the vine rows where the camera was facing downward; and other flight with flight lines perpendicular to the vine rows and with a camera angle of 45°. In both types of flights, the UAV flew at 10 m height at a speed of 2 m/s^−1^, and images were acquired with an interval of 1 s and a side lap of 60%. This flight configuration resulted in a grid of flight lines as can be seen in Figure 3.

Flights in 2020 were carried out in Graciano and Garnacha fields using a quadcopter DJI Mavic 2 Pro (DJI Inc., Shenzhen, China) equipped with a Hasselblad L1D-20c camera with a 20 MP sensor. Flight configuration concerning flight lines and sensor orientation was equal to the one used in 2019. However, and according to the preliminary analysis of 2019 imagery detailed in the results section, flight height was 15 m height, at a speed of 1.5 m/s^−1^, and images acquired with an interval of 2 s and a side lap of 75%. Image spatial resolution in nadir images was 0.20 cm and 0.38 cm for 2019 and 2020 flights, respectively.

The aerial imagery acquisition in both vineyards and years was carried out in different flights due to UAVs’ autonomy limitations. In the case of the MD4-1000 platform, the flight plan was designed using different missions that were planned taking into account the autonomy of this UAV. In 2020, one flight mission was planned for each flight type (nadir and oblique), and the Mavic platform was able of pausing the flight mission and going back to the take-off point when the battery was low, and resuming the flight after the battery replacement.

Photogrammetric processing of the aerial images was the same irrespective of whether they were collected with the Sony or the Hasselblad sensor. The 3D point clouds were generated using Agisoft Metashape Professional Edition (Agisoft LLC, St. Petersburg, Russia) version 1.7.0. The nadir and oblique images were fed together in the software, and the photogrammetric processing only required user intervention for the localization of six ground control points, georeferenced in each field the day of aerial campaign with a real time kinematic (RTK) GPS linked to a reference station from the GNSS network from the National Geographic Institute, Spain. The estimated accuracy of the GNSS-RTK system was 0.02 m in planimetry and 0.03 m in altimetry. The point clouds were stored in LASer file format (las).

### 2.3. Algorithm for Grape Cluster Detection

**Figure 4:**
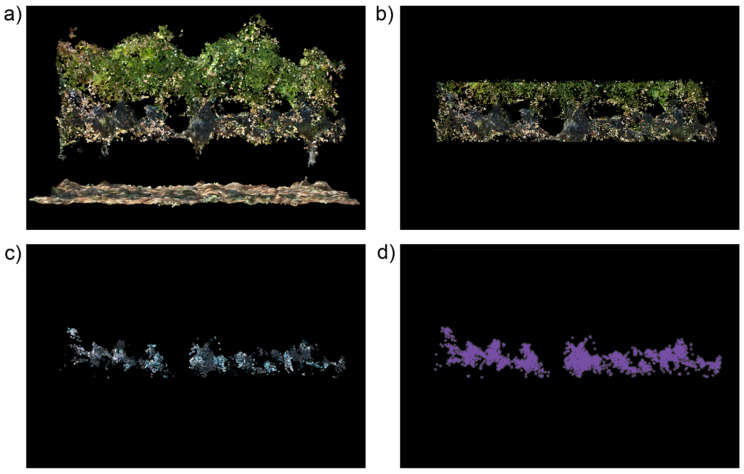
Graphical description of the main steps of the grape detection workflow (a segment of a vinerow with two sides leaf removal is used for visualization purposes): (**a**) original point cloud; (**b**) point cloud after the removal of the points with a height below 0.5 m and over 1 m; (**c**) points classified as grapes after the color filtering and the noise removal; (**d**) buffer area of the points classified as grapes.

The algorithm for grape cluster detection was created using R software 3.5.3 (R Core Team, R Foundation for Statistical Computing, Vienna, Austria, 2019) with the packages “sf” [30] and “lidR” [31]. The purpose of the algorithm is to detect the points corresponding to the grapes following two basic steps: (1) removal of the points not belonging to the area where the grape clusters grow; (2) application of a color filter to remove the points not corresponding to the grapes. In a more detailed way, the algorithm can be divided in the following steps that are automatically executed without user intervention:

1. Point cloud decimation: since the methodology has been applied to point clouds with different point densities (as a consequence of not using the same sensor and flight height configuration in 2019 and 2020), the first step was to decimate the point clouds to the average point density of the point cloud with lower resolution. The average density was calculated dividing the cloud in a square grid of 5 m side, and calculating the average of the point densities of all the squares of the grid. Point cloud decimation was performed using the *homogenize* function from *lidR* package, which produces a point cloud with a uniform density throughout the coverage area.
2. Digital elevation model (DEM) creation: the DEM of the study field was created using the cloth simulation filter (CSF) [32] as implemented in *lidR* package. Within this methodology for DEM creation, the point cloud is inverted, and a rigid simulated cloth is used to cover the inverted surface. By analyzing the interaction of the simulated cloth with the points of the 3D model, the location of the cloth nodes is used to generate a model of the terrain surface. The values of the parameters used to configure this function were: 0.5 m as threshold to consider a point belongs to the ground, as recommended by the authors of the CSF [32], 1 m as resolution of the simulated cloth, and the soil was considered almost flat in the selection of the rigidness of the simulated cloth. To speed up the DEM generation, the CSF function was applied to a voxelized version of the point cloud created with the function *voxelize_points* of *lidR* package using a resolution of 0.1 m.
3. Removal of points outside the grape clusters area: the height of the points over the terrain was calculated once the DEM was created. Because of the characteristics of the vertical shoot positioning method used to train the vines, the grape clusters were known to grow between 0.5 m and 1 m over the soil. Consequently, the points with a height below 0.5 m and over 1 m were removed from the decimated point cloud created in the first step. After the application of these height thresholds, the points corresponding to the soil, trunks, and most of the vine canopy were excluded from the next processing steps (Figure 4b).
4. Color filtering: at this step, the methodology takes advantage of the color from the points. As the mature grapes from the vine varieties growing in the studied fields are known to have a blueish color, a simple color filtering process was applied in this step. All the points having a blue value higher than their red and green values were classified as ‘grape points’. Using a mathematic expression, the points classified as grapes met the following requirements:
  B/G > 1,(1)
  B/R > 1,(2)
5. Noise removal: as the grapes grow in clusters, the purpose of this step was to remove isolated points not corresponding to the grape clusters. The function used for noise removal was the isolated voxels filter (IVF) from *lidR* package. It finds and classifies as noise points having a low amount of neighboring points in their surrounding 3 × 3 × 3 (27) voxels. The IVF was configured to classify as noise the points having less than 10 points in their surrounding 27 voxels with a side length of 0.1 m. The size of the voxels was the same used by the authors in previous works about woody crop characterization with photogrammetric point clouds [13,33], and the amount of neighboring points was fixed after some internal tests (data not published). After the execution of this step and the removal of the points classified as noise, the remaining point cloud is supposed to store only the points corresponding to the grape clusters (Figure 4c).
6. Projected area calculation: the area of the grape points projected over a vertical plane parallel to the vine rows was calculated to assess if this parameter could have a better correlation with harvest weight than the number of points classified as grapes. A radio of 1 cm, similar to the radio of a grape berry, was used to calculate a buffer around the points. These buffers were merged to avoid an overestimation of the area caused by the overlapping of the point areas (Figure 4d). The calculation of the point buffer and the resulting area were carried out with the *sf* package.

### 2.4. Data Analysis

Validation of the grape cluster detection workflow was carried out using as reference data the harvest weight registered in the harvest sampling zones described in Section 2.1. Regression analysis between the harvest weight with the number of points classified as grapes and the buffer area of these points were done in R software 3.5.3 (R Core Team, 2019) for the different fields and leaf removal treatments. These regressions were also studied for different combinations of fields to assess the transferability of the proposed methodologies among different grape varieties and flight configurations.

## 3. Results and Discussion

### 3.1. Point Cloud Generation

The combination of nadir and oblique aerial images with high overlap led to a complete reconstruction of the vines in the studied fields. Canopy, gaps in the canopy, main shoots, trunk, and grape clusters can be visually detected in the point clouds as can be seen in Figure 3. The advantages of using oblique imagery for the photogrammetric reconstruction of vertical elements such as apple tree hedgerows [34], or quarry walls [35] has been previously demonstrated. In our case, it was crucial to the reconstruction of elements that cannot be detected in nadir imagery, such as the grape clusters or the gaps in the canopy.

The Ga-20 field point cloud had an average density of 30,902 points∙m^−2^ and accurately represented the whole study area delineated in this vineyard. Gr-20 field presented a similar point density, with 35,093 points∙m^−2^, but the four sampling areas located in the easternmost part of the vineyard were not accurately reconstructed due to the flight plan was excessively adjusted to the study area limits and led to a poor overlap in this area. Consequently, these four sampling areas were excluded from the data analysis. The flight execution in Gr-19 presented some problems because this field had a zone with a slight slope area and the UAV was not able of maintaining a fixed height over the soil, which caused changes in the overlap between images and led to problems in the reconstruction of some sampling areas. These problems were the reason for the change of UAV platform and flight configuration regarding flight height and side overlap in 2020. The localization and amount of the sampling areas correctly reconstructed in Gr-19 can be seen in Figure 1 and Table 1, respectively. These areas had an average point density of 320,411 points∙m^−2^. The high difference in point density between 2019 and 2020 flights was related to the higher resolution of the sensor used in 2019 and to the lower flight height programmed in the flight plans.

As commented in the first step of the algorithm description, the point clouds were decimated to homogenize their densities and make the results comparable among point clouds from different sensor and flight configurations. As Ga-20 had the point cloud with the lowest density, the point density of Gr-19 and Gr-20 were reduced to match the density of Ga-20 (30,902 points∙m^−2^).

### 3.2. Cluster Detection

**Figure 5:**
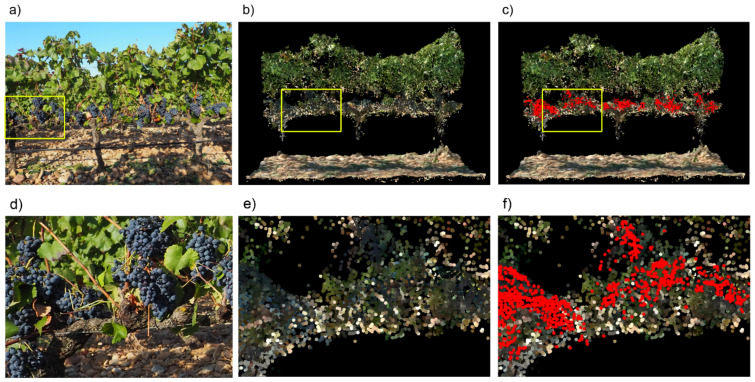
Example of the cluster detection in a vine row segment of Garnacha field with two sides leaf removal: (**a**) image of the vines in field; (**b**) segment of the point cloud showing the same vines; (**c**) same than (**b**) with cluster detected points highlighted in red color; (**d**), (**e**), and (**f**) are close ups of the areas marked by a yellow rectangle in (**a**), (**b**), and (**c**), respectively.

Figure 5 shows an example of the results of the cluster detection algorithm in a vine row segment of Garnacha field where leaf removal had been applied at both sides. The correspondence between the points detected as grapes using UAV photogrammetric point clouds (red points in Figure 5c) and the position of the grape clusters in a field photo of the same vines (Figure 5a) can be visually appreciated. From the analysis of Figure 5, it could seem that the number of points classified as grapes could be a good estimator of harvest weight. However, a study of the correlation of harvest weight with the number of points and with their projected area revealed that the last one is a better estimator. It can be seen in Table 1 that the regression coefficients are higher and more statistically significant for projected area than for the number of points. R^2^ for projected area reached values higher than 0.75 with *p* < 0.01 in three cases, while the highest R^2^ value for the number of points was 0.66, and with a higher *p*-value. Some studies [2,36] about grape detection in field images have demonstrated the existence of a high level of correlation amount the harvest weight and the number of pixels classified as grapes in the images. That is in line with the results presented in this work, since the number of pixels in a 2D image is an equivalent of the projected area of the grapes in a 3D point cloud.

**Figure 6:**
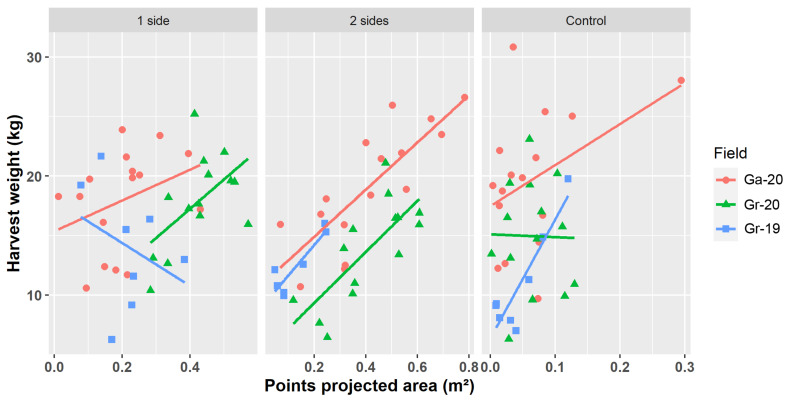
Scatter plots representing the relationship between the detected points projected area and the harvest weight for the different treatments and fields. Lines indicate the regression lines for each field.

Since the projected area of the points classified as grapes has proved to be more suitable as indicator of harvest weight, the analysis and discussion of the results will focus on this metric along the rest of the paper. The coefficients of regression between projected area and harvest weight were significant for all the fields and field combinations in the two sides leaf removal treatment. The highest R^2^ value for this treatment was 0.81 in Gr-19, followed by 0.77 in Gr-19+Ga-20 combination, and 0.63 for Ga-20. Gr-20 presented a lower R^2^ value; however, and as can be seen in Figure 6, there is a clear linear trend in the relation between projected area and harvest weight. Maybe the low R^2^ achieved could be related with the homogeneity of harvest weight along the field since the absence of extreme values uses to harden the adjustment of a linear regression. Our results are more accurate that the ones reported in [6], where an R^2^ value of 0.59 was reached applying to point clouds created with a Kinect sensor in the field and an approach similar to the one proposed in this paper to vines with a leaf removal treatment. A R^2^ of 0.93 has been reported [2], but based on an approach using field photos and requiring of a previous training for grape detection. A coefficient of regression of 0.82 between predicted and estimated yield in vines under leaf removal treatment had been reported using UAV imagery [23]. However, the cluster detection methodology used in that work is based on taking isolated photos of some sampling areas along the vineyard, while our workflow analyzes the whole point cloud representing the whole vineyard, making it possible to map yield along the entire field.

It is remarkable that the combination Gr-19+Ga-20—i.e., two fields with different grape varieties, and prospected with different sensors and flight configurations—reached a high R^2^ value (0.77). This is indicative of the robustness and transferability of the presented methodology, that have proved its accuracy in datasets with different characteristics without need of user intervention nor creation of sophisticated machine learning algorithms that require exhaustive training of the models. The combination of all datasets presented a lower R^2^ than the combination of Gr-19+Ga-20, and the regression line from Gr-20 is parallel but below the other regression lines (Figure 6). The different relation between the area of the point buffers and the harvest weight in Gr-20 could be explained by the time elapsed from the UAV flight to harvest. In Gr-19 and Ga-20 there were 8 and 6 days, respectively, between the flight and the harvest, but in Gr-20 13 days passed between these moments. Perhaps the longer time to harvest in Gr-20 caused changes in grape berries density or grape cluster compactness that altered the relation between the area of the point buffers and the harvest weight.

The regression coefficients between points of projected area and harvest weight were low and in some cases not statistically significant for the control treatment (Table 1). This is in agreement with other works where the influence of leaf occlusion has been reported as a major challenge in fruit detection in general [37], and also on grape detection in both on-ground images [6] and UAV images [23]. The only exception for this general rule was Gr-19 in the vines with no leaf removal, which registered the highest R^2^ value (0.82) among all the fields and treatments. This exception could be related to the fact that, although the point cloud was decimated, the original point cloud was created with images with very high spatial resolution (images from Gr-19 had a spatial resolution of 0.2 cm), and this could have facilitated a more detailed reconstruction of small parts of the grape clusters.

In the one side leaf removal treatment, only the leaves from the east side of the vines were removed, which caused the grape clusters growing in the west side to be occluded or only partially visible. Consequently, vines with most of the grapes growing in the west side would present a low value of projected area of grape points, while vines with more grape clusters in the east side would have higher values of projected areas. This fact led to the lack of a linear relationship between points projected area and harvest weight (Figure 6), causing the lowest R^2^ values and the absence of statistical significance reached in the one side leaf removal treatment (Table 1).

### 3.3. Applicability of Presented Methodology and Future Research

The methodology presented in this paper can be applied in one working day with the following time distribution: less than 2 h for the UAV flights, around 16 h for point cloud generation, and almost 1 h for point cloud analysis. From this time, the user intervention is only needed for executing the UAV flights, and for the localization of the GCPs in the point cloud generation step, which requires about 20 min. Apart from the efficiency, the methodology has other important advantages that make it suitable for the detection of grape clusters and yield prediction in vines under two sides leaf removal treatment:

1. The grape clusters detection algorithm has been developed by using inexpensive UAV (flights in 2020 were carried out with an UAV about 1500 USD) and sensor, and R free software, all of them are considered low-cost technology. These accessible tools constitute an affordable, cost-effective, and easily repeatable technology for a wide range of grape growers.
2. It works with point clouds representing the entire vineyard. This fact makes possible the estimation of harvest weight from all the vines in the parcel at the right moment using a nondestructive method without limiting the yield forecasting to some sampling points, which would hinder the detection of the spatial variability of the vineyard production.
3. Since the points classified as grapes have coordinates, the methodology presented allows the generation of yield maps if it is combined with procedures for individual vine detection or division of vine rows in segments like the ones developed by [38] and [39], respectively. The harvest estimation maps generated near the harvest time allow the zoning of harvest operations, reserving the most adequate yield levels, e.g., for premium wine production, as suggested by Ballesteros et al. [25].
4. It does not need the creation of a training dataset for grape cluster detection, unlike most of the previously reported methodologies for fruit detection in field images [40,41,42].
5. It overcomes the problem of the on-ground image analysis related to the need of driving the image acquisition platform through the entire vineyard acquiring images from both sides of the vinerows, which in some cases is hard to achieve due to challenging conditions (e.g., slope, wet soil).

Although the workflow presented in this paper has showed high potential for yield estimation in vineyards with ripe red wine grapes, it has some limitations. It works sufficiently well only in vines with two-sides leaf removal, a canopy management practice that, although common in some wine-growing regions, is not feasible in other areas due to possible adverse effects, such as sunburn or overripening of grapes. Another limitation could be the low flight altitude used in the flights. This would imply more UAV flights per day to apply the workflow to large vineyards because the current batteries for UAV’s autonomy not for lack of computational requirements. In any case, the use of UAV-imagery would be more profitable than the traditional yield forecasting by using on-ground measurements in some sampling points spending more time and personnel resources. For these reasons, future research will focus on determining the optimal canopy coverage and sensor resolution for grape detection, trying to emulate the results achieved in the control treatment in Gr-19. Further research will also try to confirm the robustness of the presented workflow by including more red grape varieties apart from Graciano and Garnacha, and to study the influence on yield prediction of the time elapsed between the UAV flight and the harvest. Since white grapes cannot be detected using the proposed methodology due to the similar reflectance of grapes and leaves when working with RGB conventional cameras, future research will also address the detection of white grapes using a hyperspectral sensor to search for wavelengths allowing an accurate and robust discrimination of grapes and leaves.

## 4. Conclusions

An unsupervised and automatic method for grape cluster detection by the classification of UAV-3D-photogrammetric point clouds by color indices has been developed using data from two locations and years, and two red grape varieties. It has been evaluated under different leaf removal treatments to assess the influence of leaf occlusion, and it has proved to be robust and accurate at the whole vineyard scale in vines with two sides leaf removal treatments, although promising results have also been achieved in vines with untouched full canopies. Furthermore, to the best of authors’ knowledge, this is the first time that UAV photogrammetric point clouds have been used for grape cluster detection. These results demonstrate the potential for linking low-cost UAVs and sensors, and free software for developing a methodology for yield estimation in red grape vineyards, opening the door to the creation of yield forecasting maps that will have an important and positive impact in the management of harvest and wine production operations, easing the implementation of precision viticulture and digitizing-related strategies.

## Figures and Tables

**Table 1:** Correlation coefficients between harvest weight and the studied variables for the different leaf removal treatments and fields.

Treatment	Field	Sampling Areas	R^2^ for Detected Points	R^2^ for Projected Area of Detected Points
One side leaf removal	Gr-19	8	0.09 *ns*	0.11 *ns*
	Gr-20	14	0.02 *ns*	0.31 *
	Ga-20	16	0.05 *ns*	0.11 *ns*
	Gr-19 + Ga-20	24	0.05 *ns*	0.01 *ns*
	All	38	0.00 *ns*	0.06 *ns*
Two sides leaf removal	Gr-19	7	0.57 *ns*	0.81 ***
	Gr-20	14	0.55 **	0.56 **
	Ga-20	16	0.48 **	0.63 ***
	Gr-19 + Ga-20	23	0.59 ***	0.77 ***
	All	37	0.35 ***	0.52 ***
Control	Gr-19	8	0.66 *	0.82 **
	Gr-20	14	0.01 *ns*	0.00 *ns*
	Ga-20	16	0.18 *ns*	0.17 *ns*
	Gr-19 + Ga-20	24	0.11 *ns*	0.22 *
	All	38	0.05 *ns*	0.13 *

*ns* not significant; * *p* < 0.05; ** *p* < 0.01; *** *p* < 0.001.

## Data Availability

The datasets generated during the current study are available from the corresponding author on reasonable request.
